# Analysis of quantitative [I-123] mIBG SPECT/CT in a phantom and in patients with neuroblastoma

**DOI:** 10.1186/s40658-019-0267-6

**Published:** 2019-12-30

**Authors:** Samuel L. Brady, Barry L. Shulkin

**Affiliations:** 1 Department of Radiology, Cincinnati Children’s Hospital Medical Center, University of Cincinnati, Cincinnati, OH 45229 USA; 2 Department of Diagnostic Imaging MS 220, St. Jude Children’s Research Hospital, 262 Danny Thomas Place, Memphis, TN 38105-3678 USA

**Keywords:** mIBG, Nuclear medicine, Neuroblastoma, Pediatrics

## Abstract

**Purpose:**

To determine the accuracy of quantitative SPECT, intersystem and interpatient standardized uptake value (SUV) calculation consistency for a manufacturer-independent quantitative SPECT/CT reconstruction algorithm, and the range of SUVs of normal and neoplastic tissue.

**Methods:**

A NEMA body phantom with 6 spheres (ranging 10–37 mm) was filled with a known activity-to-volume ratio and used to determine the contrast recovery coefficient (CRC) for each visible sphere, and the measured SUV accuracy of those spheres and background water solution. One hundred eleven 123I-metaiodobenzylguanidine ([I-123] mIBG) SPECT/CT examinations from 43 patients were reconstructed using SUV SPECT® (HERMES Medical Solutions Inc.); 42 examinations were acquired using a GE Infinia Hawkeye 4 SPECT/CT, and 69 were acquired on a Siemens Symbia Intevo SPECT/CT. Inter scanner SUV analysis of 9 regions of normal [I-123] mIBG tissue uptake was conducted. Intrapatient mean SUV variability was calculated by measuring normal liver uptake within patients scanned on both cameras. The intensity of uptake by neoplastic tissue in the images was quantified using maximum SUV and, if present, compared over time.

**Results:**

The phantom results of the visible spheres and background resulted in accuracy calculations better than 5–10% with CRC correction. Interscanner SUV variability showed no statistical difference (average *p* value 0.559; range 0.066–1.0) among the 9 normal tissues analyzed. Intrapatient liver mean SUV varied ≤ 16% as calculated for 28 patients (87 examinations) studied on both scanners. In one patient, a thoracic tumor evaluated over 10 time points (18 months) underwent a 74% (3.1/12.0) reduction in maximum SUV with treatment.

**Conclusion:**

The results demonstrate quantitative accuracy to better than 10%, and both consistent SUV calculation between 2 different SPECT/CT scanners for 9 tissues, and low intrapatient measurement variability for quantitative SPECT/CT analysis in a pediatric population with neuroblastoma. Quantitative SPECT/CT offers the opportunity for objective analysis of tumor response using [I-123] mIBG by normalizing the uptake to injected dose and patient weight, as is done for PET.

## Introduction

Many conventional nuclear medicine studies were developed or adapted to provide quantitative information about a functional process. These examinations include gastric emptying, differential renal function and tracer excretion, cardiac ejection fraction, radioiodine uptake, differential lung perfusion, and gallbladder ejection fraction. However, single-photon emission computed tomography (SPECT) has traditionally been regarded as a non-quantitative imaging modality primarily because of the lack of correction for photon absorption and photon scatter.

With the pairing of computed tomography (CT) and SPECT systems (SPECT/CT), photon absorption is accounted for in SPECT imaging, and similar to positron emission tomography (PET) and PET/CT, provides the basis for rendering SPECT imaging quantitative. Additional advances in reconstruction algorithms have been made available to render SPECT/CT quantitative, namely correcting for partial volume resolution losses and scatter contamination of the main photon peak, and converting pixel values (kBq cts^− 1^) to express in vivo radioactivity concentrations (Bq cm^− 3^) [1–5]. The collimator-detector response (i.e., resolution loss) [6, 7] and scatter correction, based on accelerated Monte Carlo scatter compensation [8–10], are implemented for each scanner and collimator pair, as previously validated, and are provided as an inherent correction within the SPECT post processing software; however, to calibrate the volumetric SPECT image set to express values of radiotracer concentration, a conversion factor [5, 11, 12] is derived by imaging a uniform water phantom of known water volume that contains a known amount of radioisotope activity. The latter is derived by the user for input to the reconstruction software, similar in fashion to the routine dose calibrator quality control performed on PET/CT scanners. With the development of software corrections to render SPECT/CT quantitative, region-of-interest (ROI) measurements can be taken from a SPECT image and used to calculate a standardized uptake value (SUV) in similar fashion as PET imaging has been used for 2-deoxy-2-[fluorine-18]fluoro-D-glucose ([F-18] FDG) analysis—by normalizing the pixel activity concentration (Bq cm^− 3^) from the image with decay-corrected, patient-injected activity and patient weight (Bq g^− 1^) [13, 14].

As Bailey and Willowson [3] have described, quantitative SPECT imaging has five potential advantages over PET. First, longer lived half-lives are more in-line with biological half-lives, and allow for easier transport of the radiotracers, thus decoupling the imaging site with cyclotrons. Second, SPECT imaging includes a larger variety of radiotracer options with which to explore a variety of clinical indications. Third, SPECT imaging enables single examination for exploration of complimentary or competing biological systems. Fourth, the general costs to purchase and operate a gamma camera are lower. And fifth, nuclear medicine departments using SPECT imaging technology are widespread and well established around the world.

Since 2008, several studies investigating clinical use of radiotracers for quantitative SPECT/CT have shown a list of wide-ranging clinical indications that can be explored quantitatively by using SPECT/CT (e.g., brain tumor analysis with [Tl-201], bone analysis using [Tc-99 m] diphosphono-propanedicarboxylic acid (DPD), lung perfusion [Tc-99 m] macroaggregated albumin (MAA), ventricular ejection fraction with [Tc-99 m] radiolabeled erythrocytes) [3, 15, 16].

The purpose of this study was to use SPECT/CT imaging to quantitatively characterize various sites of normal and abnormal uptake of 123I-meta-iodobenzylguanidine ([I-123] mIBG) in patients with neuroblastoma. The objectives were to (1) characterize the accuracy of a manufacturer-independent quantitative SPECT/CT reconstruction algorithm, (2) determine intersystem variability of two different manufacturer’s SPECT/CT scanners, (3) determine intrapatient SUV calculation consistency from both SPECT/CT scanners, (4) determine the range of SUV values for uptake in normal and neoplastic tissue, and (5) to obtain preliminary data on the potential utility of serial quantitative measurements of tumor uptake in patients undergoing cytoreductive treatment.

## Materials and methods

### Patient study design

This study was approved by our institutional review board, and the need for written informed consent was waived. All data were and managed in compliance with the Health Insurance Portability and Accountability Act. A total of 111 [I-123] mIBG SPECT/CT examinations were reconstructed by using SUV SPECT® (HERMES Medical Solutions Inc., Montreal Quebec). The 111 examinations were obtained from the local archival database for the months April 2015 through March 2016. Of the examinations described in this study, the first 42 were acquired using a GE Infinia Hawkeye 4 (hereafter referred to as “Infinia”), April through July 2015, and the remaining 69, using a Siemens Symbia Intevo (hereafter referred to as “Symbia”), August 2015 through March 2016. Image analysis of two patients was extended through July 2016 for inclusion in Figs. 2 and 4 .

Per the institutional standard imaging protocol, each patient received [I-123] mIBG intravenously [370 MBq (10 mCi) per 1.73 m^2^ body surface area, minimum 74 MBq (2 mCi) and maximum 370 MBq (10 mCi)]. To block [I-123] uptake in the thyroid, a saturated solution of potassium iodide drops (1 drop three times daily) was administered 24 h prior to [I-123] mIBG injection and continued for a total of 3 days. Approximately 24 h post [I-123] mIBG administration, whole-body and spot images were acquired followed by SPECT/CT images. The SPECT field of view was determined based on the technologist’s consultation with the nuclear medicine physicians but generally included the neck, chest, abdomen, and pelvis; imaging of the cranium and extremities was not included in this analysis due to a paucity of data. SPECT acquisition parameters for both scanners included 180-degree configuration for a total of 96 views at 35 s/stop with a 128 × 128 matrix. The Infinia used medium-energy (ME) collimators for both planar and SPECT images, and the Symbia used low-energy high-resolution collimators (LEHR). Directly following the SPECT acquisition, a CT series was acquired for attenuation correction and anatomical co-localization purposes. For the Infinia, the CT acquisition parameters were 120 kV, 59 mAs, 2.6-s rotation time, no zoom, and collimation of 4 × 5 mm. Images were reconstructed using filtered back projection (FBP) with a standard soft tissue reconstruction kernel. The average examination volume-computed tomography dose index (CTDIvol) for the Hawkeye was 13.1 mGy (assuming a 32-cm diameter CTDI phantom). For the Symbia, the CT acquisition parameters were 80 kV, 30 quality ref mAs, 0.6-s rotation time, pitch of 0.8, no zoom, and collimation of 16 × 1.2 mm. Standard images were reconstructed by using FBP with a soft tissue reconstruction kernel (I31s). The average examination CTDIvol for the Symbia was 0.69 mGy (assuming a 32-cm diameter CTDI phantom).

### Patient image reconstruction and SUV calibration

To render the Infinia and Symbia acquired SPECT images quantitative, each patient’s examination SPECT and CT image sets were reconstructed using HERMES’ SUV SPECT® software. The SPECT images were reprocessed using 5 iterations and 16 subsets of an ordered subset expectation maximization (OSEM) reconstruction followed by a series of corrections applied to the image raw data to correct for count losses due to attenuation from the patient (using the CT image set), partial volume resolution loss, and photon scattering contamination of the main photon peak. Following the SPECT reconstruction, a conversion factor (units: kBq cts^− 1^) was applied to convert pixel values (units: cts cm^− 3^) to represent a value of radioactivity concentration (units: kBq cm^− 3^). The conversion factor was derived using a Jaszczak cylinder phantom (without any inserts) filled with a known water volume. The water phantom was prepared with an activity of 111 MBq (3 mCi) of [I-123] mIBG calibrated using a Capintec CRC-15R dose calibrator; the dose calibrator accuracy was calculated to be better than 1.5% (as calculated for 8 different radioisotopes including [I-123]). The conversion factor was derived based on 111 MBq (3 mCi) to keep the count rate on the scanner < 20 kcts s^− 1^, per HERMES instructions. SPECT/CT images were acquired and processed within the HERMES Hybrid Recon domain to produce the conversion factor. A separate conversion factor was calculated for both the Infinia scanner, using ME collimators, and the Symbia scanner, using LEHR collimators. For each patient’s examination, the administered radiopharmaceutical dose (determined by subtracting postinjection activity from preinjection activity in the injection syringe), time of injection, patient’s weight, patient’s height, and scan start date/time were entered into the software.

### Phantom study

Quantitative accuracy was measured using a National Electrical manufacturers Association (NEMA) International Electrotechnical Commission (IEC) body phantom with six fillable spherical and one lung insert. The lung insert (measured 50 mm outer dia.) was placed at the center of the phantom and was filled with low density Styrofoam balls (average *ρ =* 0.3 ± 0.1 g cm^− 3^; μ_lung_ ~ 0.026 cm^− 1^) and air to mimic lung attenuation. Six spherical inserts (inner diameter 10, 13, 17, 22, 28, and 37 mm) were filled with a mixture of [I-123] mIBG and water.

The phantom was prepared using an ~ 3:1 ratio of water to [I-123] mIBG. A 3000 cm^− 3^ volume of water was prepared with a calibrated net activity of 106.2 MBq (2.9 mCi) which led to an activity-by-volume of 35.4 kBq cm^− 3^. Radioactive water was drawn from the 3000 cm^3^ solution and used to fill the six spherical inserts: 10-mm sphere received 0.5 cm^3^, 13-mm sphere received 1.0 cm^3^, 17-mm sphere received 2.8 cm^3^, 22-mm sphere received 5.8 cm^3^, 28-mm sphere received 11.7 cm^3^, and 37 mm received 29 cm^3^ of radioactive water. The body phantom, with inserts in place, was filled to full capacity (9650 cm^3^). The phantom preparation-to-scan interval was approximately 2 h. The phantom was scanned using the same acquisition techniques described in section II.A using only the Symbia.

Once acquired, the images from the Symbia were processed using HERMES SUV SPECT® for quantitation. SUV values were measured by placing ROIs within the area of the observed radioactive spheres on the images using the following formulation:

1 $$ SUV=\frac{A_v}{\frac{A}{w}}, $$

where *A*_*v*_ is the activity-by-volume measured with the ROI, *A* is the net activity measured by the dose calibrator, and *w* is the weight of the phantom/patient entered at the scanner console at the time of the study; the weight of the phantom with water was 16 kg and used for the SUV calculation. Units of SUV calculated in the phantom were g cm^− 3^; note SUV values calculated in tissue (i.e., patients) have no units because the activity-by-volume is measured using an ROI over tissue that has a mass density of 1 g cm^− 3^, thus, the activity-by-volume is converted to tissue tracer activity by dividing *A*_*v*_ by g cm^− 3^ to yield units of kBq g^− 1^. Additionally, ROIs were placed throughout the phantom volume to measure activity in the background water. The ROI measurements from the spheres and the background were combined to calculate the contrast recovery coefficient (CRC):

2 $$ CRC=\frac{\frac{SUV_{\mathrm{sphere}}}{SUV_{\mathrm{bkgd}}}-1}{\frac{A_{\mathrm{sphere}}}{A_{\mathrm{bkgd}}}-1}, $$

where *SUV*_sphere_ and *SUV*_bkgd_ are the measured sphere and average background SUV (g cm^− 3^) respectively, and *A*_sphere_ and *A*_bkgd_ are the known sphere and background activity-by-volume (kBq cm^− 3^), respectively. The measured SUV values for the spheres and background were used to calculate a predicted sphere activity-by-volume (${A}_v^{\mathrm{measured}}$) using Eq. 1 and corrected for 1-CRC loss of counts due to system resolution limits:

3 $$ {A}_v^{\mathrm{measured}}= SUV\bullet \frac{A}{w}\bullet \left[1+\left(1- CRC\right)\right]. $$

The ${A}_v^{\mathrm{measured}}$ was compared with the actual activity-by-volume calculated for the fillable spheres 35.4 kBq cm^− 3^ and for the background 11.0 kBq cm^− 3^.

### Study analysis

Each examination was analyzed for interscanner SUV reproducibility for 9 regions of normal [I-123] mIBG tissue uptake, namely left/right parotid glands, left/right submandibular glands, left ventricle of the heart, liver, left/right adrenal glands, and the bladder. Additionally, neoplastic tissue, when present in the examination data, was quantified by using SUVs and explored over time. Regions of interest were drawn over the SPECT images and overlaid on the CT for anatomic confirmation. An analysis of the intrapatient mean SUV variability was calculated by measuring normal liver uptake for patients scanned on both scanners. Intrapatient variability was measured for 28 patients with two or more studies at different time points: two studies (*n* = 6), three studies (*n* = 14), four studies (*n* = 5), five studies (*n* = 1), six studies (*n* = 1), and ten studies (*n* = 1).

Descriptive statistics were used to describe the data. A two-tailed Mann-Whitney *U*-test (significance level equal to 0.05) was used to compare SUVs measured in the normal tissues. Statistical calculations were performed using PRISM (GraphPad, La Jolla, CA).

### Patient demographics

A total of 111 examinations obtained over 12 months (April 2015 through March 2016) were analyzed for this study, with 43 unique patients imaged (22 boys, 60 studies; 21 girls, 51 studies; number of studies per patient, 1–10). The patients’ median age was 3.0 years (range 0.8 to 17 years); median weight, 16.1 kg (range 9.4 to 90.9); median height, 98.1 cm (range 72 to 174 cm); median BSA, 0.7 m^2^ (range 0.4 to 2.1 m^2^); and median radiopharmaceutical dose of [I-123] mIBG, 162.8 MBq (range 107.3 to 429.2 MBq) [4.4 mCi (range 2.9 to 11.6 mCi)]. Three patients received radiopharmaceutical doses above 370 MBq (10 mCi) after consultation with the nuclear medicine physician because of their age, weight, and BSA concentration at the time of examination.

## Results

### Phantom study

**Fig. 1:**
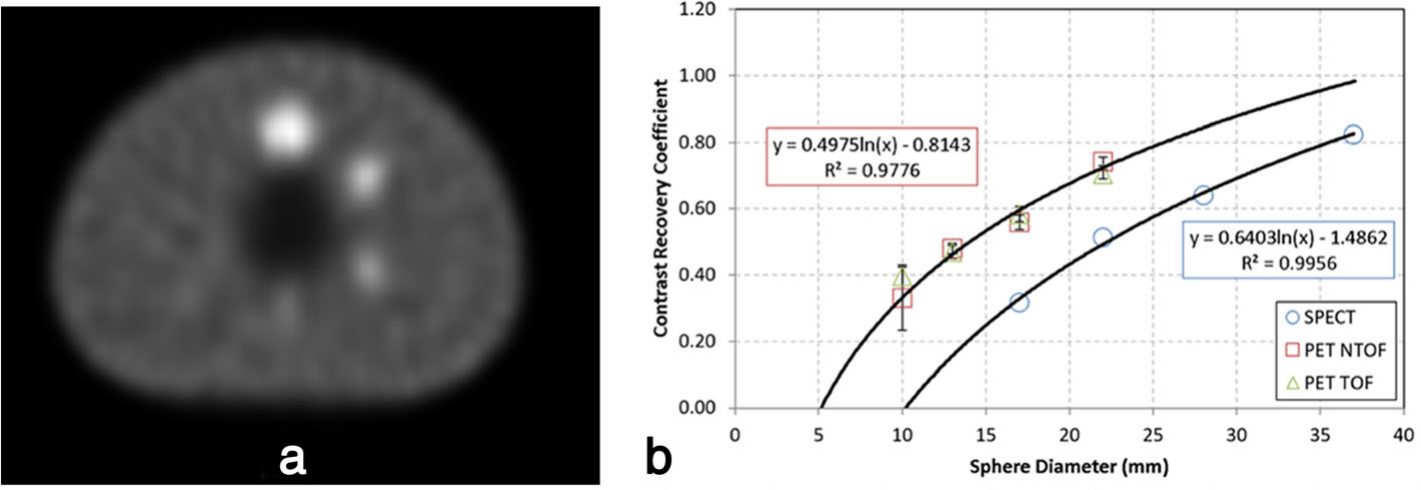
Accuracy of quantitative SPECT. **a** NEMA body phantom imaged with a 3:1 ratio of [I-123] using the Symbia. **b** The SPECT contrast recovery coefficient (CRC) was calculated for the four visible spheres; for reference, PET CRC values are provided (GE Discovery 690 VCT)

The phantom was imaged to measure accuracy of quantitative SPECT (Fig. 1a). SPECT CRC values for the four visible spheres were calculated: 0.32 (17-mm sphere), 0.51 (22-mm sphere), 0.64 (28-mm sphere), and 0.82 (37-mm sphere) (Fig. 1b); the SPECT CRC values were compared with [F-18] FDG PET CRC values, as measured on a GE Discovery 690 VCT for reference. The results of ${A}_v^{\mathrm{measured}}$ for SPECT, as calculated using Eq. 3 for the four visible spheres are 33.5 kBq cm^− 3^ (17-mm sphere), 37.2 kBq cm^− 3^ (22-mm sphere), 38.5 kBq cm^− 3^ (28-mm sphere), and 38.9 kBq cm^− 3^ (37-mm sphere), and for background is 11.7 kBq cm^− 3^. These results agreed with the actual activity-by-volume for the spheres of 35.4 kBq cm^− 3^ and for the background 11.0 kBq cm^− 3^ to better than − 5%, 5%, 9%, 10%, and 6%, respectively (where the negative value represented an underestimation).

### Intersystem comparison with normal tissue uptake SUVs

**Fig. 2:**
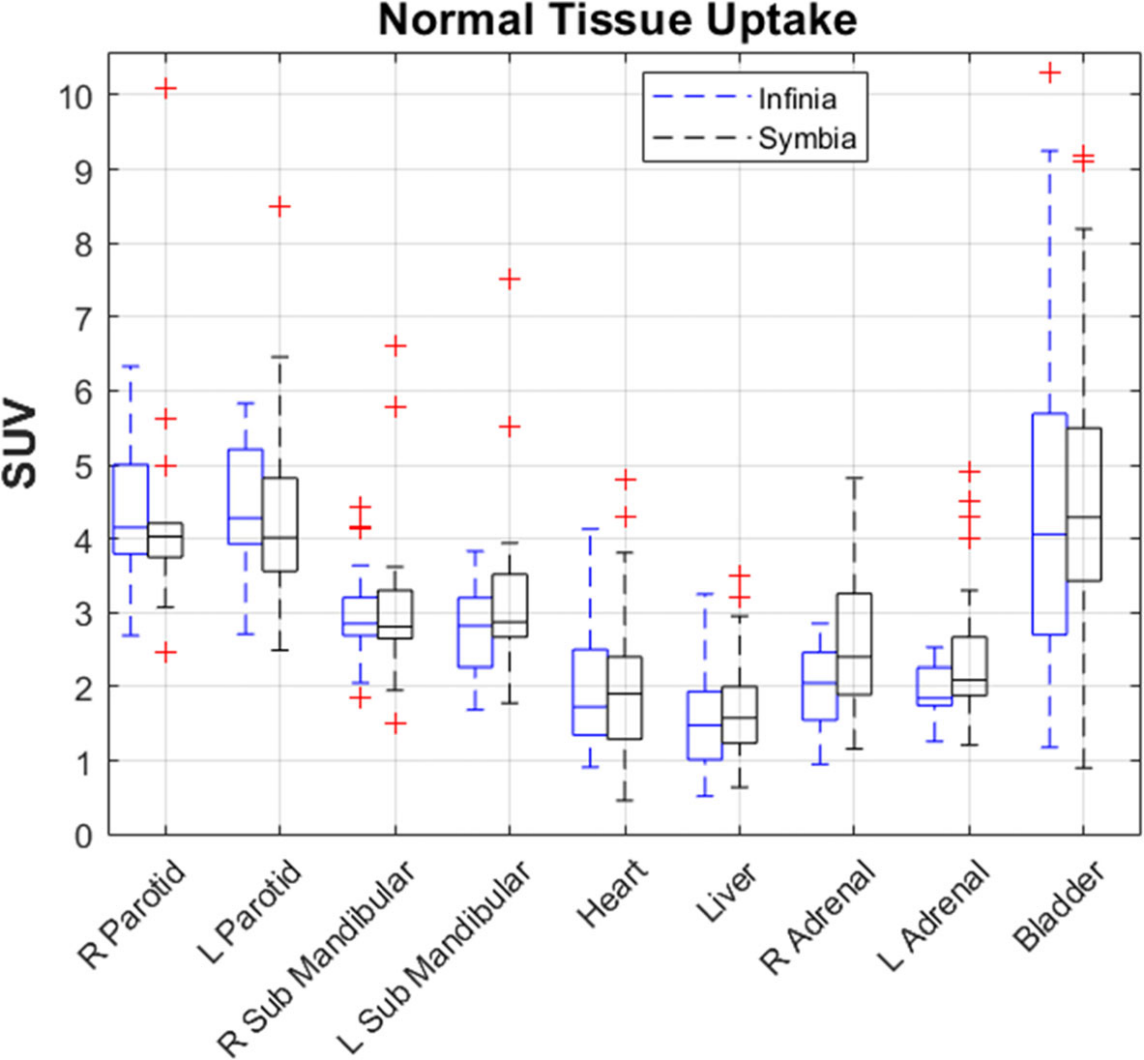
Interscanner SUV variability was plotted with outlier data (+) for 9 normal tissues imaged on an Infinia (left box plots-blue color) and on a Symbia (right box plots-black color). The SUVs values of the scanners were not significantly different

Normal tissue uptake SUVs were measured, and interquartile ranges (IQRs) were calculated. The values showed consistency between the Infinia (mean IQR of 1.1; range 0.4–2.8) and the Symbia (mean IQR of 1.0; range 0.4–2.0) (Fig. 2). Mean SUVs (± 1 SD) for the 9 tissues are reported in Table 1; the mean values of the Infinia and Symbia scanners were not significantly different (average *p* value of 0.559; range 0.066–1.0), (Table 1).

**Table 1:** Mean SUV values for normal tissue uptake, as measured on the Symbia and Infinia, are provided

Normal tissue	Infinia	*N*	Symbia	*N*	*U* score	*p* value
R. Parotid	4.4 ± 1.5	16	4.4 ± 1.8	14	92 (64)	0.418
L. Parotid	4.4 ± 1.3	16	4.4 ± 1.6	14	95 (64)	0.490
R. Sub. Mandibular	3.0 ± 0.7	19	3.2 ± 1.2	18	169 (106)	0.952
L. Sub. Mandibular	2.8 ± 0.6	19	3.3 ± 1.3	18	134 (99)	0.368
Heart (L. Ventricle)	2.0 ± 0.3	33	2.0 ± 0.8	64	1056	1
Liver	1.5 ± 0.6	39	1.6 ± 0.5	63	1078	0.294
R. Adrenal	2.0 ± 0.3	14	2.5 ± 0.9	23	113	0.136
L. Adrenal	1.9 ± 0.2	6	2.4 ± 0.9	43	140	0.091
Bladder	4.4 ± 1.5	35	4.4 ± 1.9	50	843	0.779

The number (*N*) of instances for each organ measurement is provided per machine. The Mann-Whitney *U*-test *U* score and (critical value) along with *Z*-score *p* values are provided; significance for Mann-Whitney *U*-test results that were determined to be normal was based on Z-score *p* values only, i.e., no critical value was provided

### Intrapatient variability

The overall maximum (mean SUV = 3.2) and minimum (mean SUV = 0.5) liver SUV percentage is calculated to be no greater than 16% (0.5/3.2) for the 28 patients who underwent imaging on both cameras. The average mean liver SUV of the 28 patients is 1.6 with a calculated variance of the mean 0.1.

### Neoplastic uptake SUV values

**Fig. 3:**
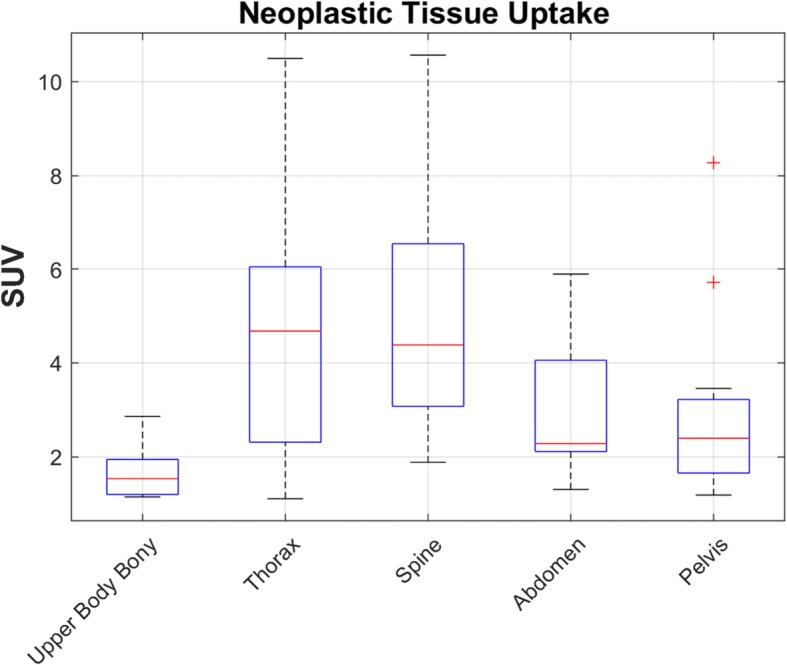
Avid neoplastic tissue was quantified by SUV for images acquired on the Infinia and the Symbia combined

Neoplastic tissue was identified in 39 examinations (16 patients) from the images obtained on the Infinia and in 36 examinations (25 patients) on the Symbia. The neoplastic uptake was categorized by location, namely upper body bony (i.e., left/right scapula, left/right humerus, and sternum), vertebral spine (thoracic, lumbar, sacrum), pelvis (i.e., left/right ilium, and left/right hip), thorax (i.e., left/right para vertebral left/right ribs, soft tissue chest), and abdomen (i.e., abdomen soft tissue, midline, left/right adrenal, retroperitoneal). In all, 75 sites of avid neoplastic tissue were quantified using SUV for the cases in which the patients were imaged on the Infinia and the Symbia (Fig. 3). The range of SUV values measured for each anatomical location of neoplastic tissue had a relative minimum (~ SUV of 1) and maximum (~ SUV of 10) range for neoplastic uptake over a time greater than a year.

## Discussion

The objectives of this study were to characterize the accuracy of a manufacturer-independent quantitative SPECT/CT reconstruction algorithm, determine intersystem and intrapatient SUV calculation consistency, and to determine a range of SUV values for uptake in normal and neoplastic tissue. The accuracy of the quantitative SPECT/CT reconstruction algorithm, as determined by a dose calibrator standard, agreed to better than 5% for objects ≥ 17 mm in diameter, which correlates with CRC values > 32%. Similarly, the reconstruction algorithm agreed to better than 6% for the background water solution. In a previous publication investigating [I-123] for brain SPECT [17], Monte Carlo simulations demonstrated quantitative accuracy overestimation to better than 10%. In another publication [18], Koral measured and applied CRC corrections, similar to this study, to correct for the loss of contrast in the image due to the resolution limits of the SPECT scanner. In that work, the quantitative calculation of [I-131] SPECT of tumor volumes ranging from 7 to 135 cm^3^, the quantitative accuracy underestimated true activity in spite of the CRC correction for the small tumor volumes (7 cm^3^) on the order of − 23.8%, but agreed to better than 4.3–11.2% for larger volumes (16 to 135 cm^3^). In other publications, quantitative SPECT accuracy was demonstrated for [In-111] between 2 and 12% (6 to 20 cm^3^ spheres) [19], [Ho-166] between 1 and 13% (220 cm^3^ cylinder) [20], and [Tc-99 m] between 4 and 11% (0.5–40 cm^3^ objects) [3, 21, 22].

In this study, the CRC was not calculated for the Infinia as a point of comparison to the Symbia; that said, based on the intra- and inter- patient SUV calculations made in this study, which were generally consistent between scanners, we speculate that the CRC measurements would most likely be comparable from scanner-to-scanner. With collimator specific corrections applied, the SUV value calculated on both scanners demonstrated a fairly robust agreement of interscanner SUV accuracy. Thus, assuming most variables being equal (e.g., phantom activity, similar ROI size, similar collimator-to-phantom distance, similar number of counts per projection), the overall CRC should be similar between SPECT scanners, with the major limiting factor being the resolution of the scanner and collimator pair, and being able to accurately reconstruct and resolve the smaller diameter target insert(s) to perform SUV, and consequently, CRC calculation(s).

When comparing interscanner uptake patterns of [I-123] mIBG in normal tissue between the Infinia and the Symbia, similar concentration levels (i.e., mean SUV values), IQR values, and patterns of uptake in the various tissues were demonstrated. SUV in the parotid glands was higher than in the submandibular glands, a pattern of uptake in agreement with that in a previous publication [23] involving a semi-quantitative investigation of salivary gland uptake using [Tc-99 m] pertechnetate. Additionally, the mean SUV of the liver was relatively lower than that of all other tissues, and adrenal glands demonstrated balanced (i.e., left/right) mean SUV values. Although overall uptake patterns were demonstrated in normal tissue, a wider spread of data (i.e., minimum to maximum) was seen in the Symbia than in the Infinia. The difference in the spread in data may be partially attributed to the use of LEHR collimators on the Siemens scanner rather than the ME collimators used on the GE scanner. With the higher penetration energy of the [I-123] electron capture and subsequent gamma ray emission from [Te-123] (main photon peak at 159 keV), greater amounts of scatter and penetration occurred, potentially leading to greater variability in uptake but not enough to render the SUV measurements from the Symbia statistically different than those of the Infinia (average *p* value of 0.559). Additionally, the spread of SUV data is attributed to the Symbia scanner having better CT resolution than the Infinia, which led to greater confidence in assigning lower level activity to a specific region measured by ROI. The SUV values in Table 1 may provide a baseline range for [I-123] mIBG in normal tissue.

**Fig. 4:**
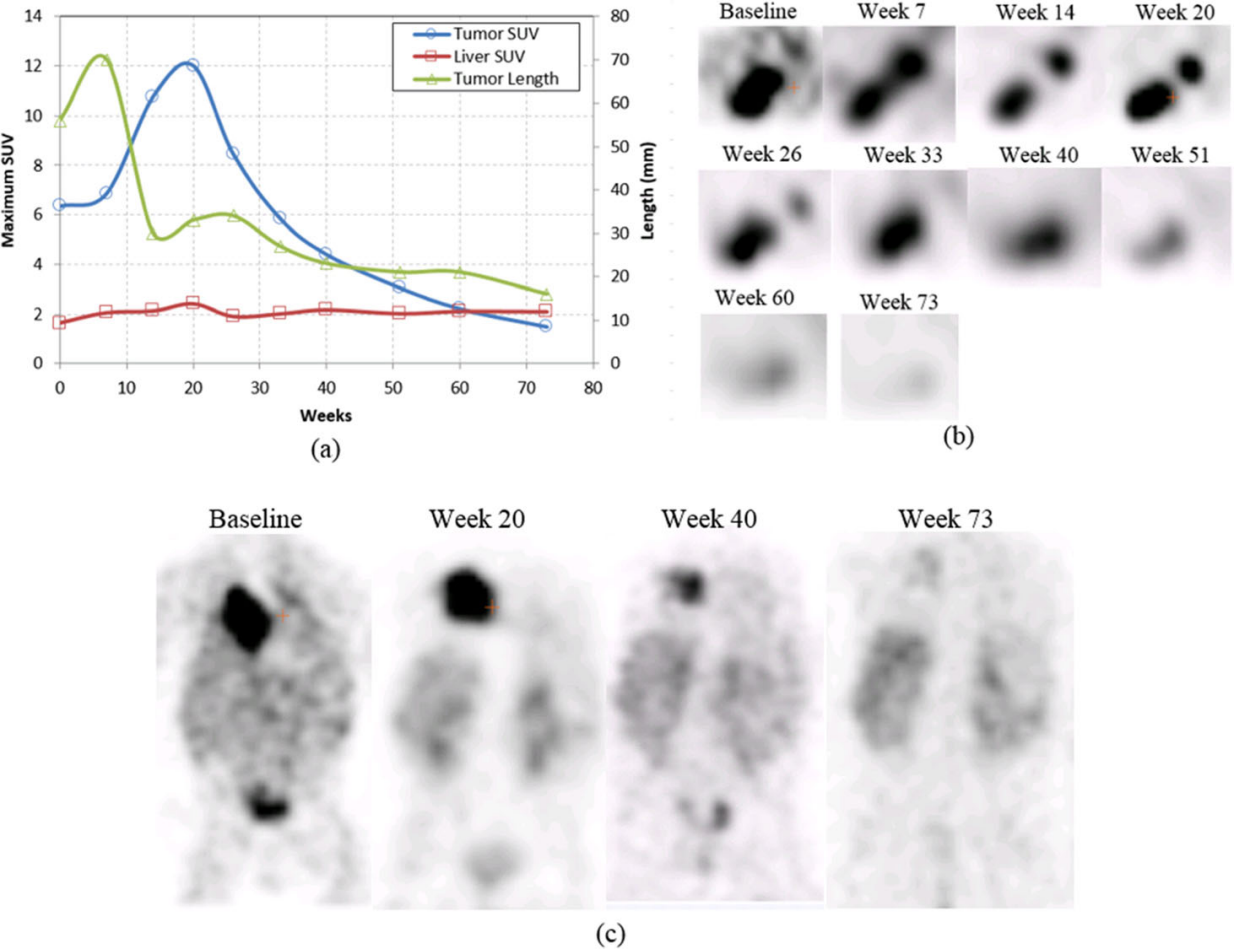
Example of quantitative SPECT analysis of a patient undergoing treatment for 1 year and 4 months. **a** The primary paraspinal thoracic tumor was quantified by determining maximum SUV value and compared with long-axis length measurements of the larger presentation of the multifocal tumor. An SUV measurement was performed in the liver to provide a baseline SUV value for normal background uptake. **b** Transaxial reconstructed images were captured at 10 time points, with **c** selected coronal reformats shown to provide general context of the tumor size, avidity, and response to therapy compared to whole-body uptake

[I-123] mIBG uptake in neoplastic tissue varied greatly, with no discernable pattern according to anatomical region. Although no consistency in mean uptake was demonstrated, overall uptake variance was consistent between both scanners over a time period greater than a year. A quantitative analysis of SPECT [I-123] mIBG may provide additional clinically relevant information when analyzed longitudinally, as demonstrated in Fig. 4, which shows a 7-month-old infant with an [I-123] mIBG-avid right chest paraspinal neuroblastoma. Tumor uptake was evaluated over 10 time points while patient underwent treatment, and a retrospective quantitative analysis of the primary tumor demonstrated a progressive reduction in maximum SUV. Imaging, and subsequent SPECT SUV analysis, was performed on the Infinia from baseline through week 20; from week 26 through 73, all imaging and SUV analysis was performed on the Symbia. SUVs of the liver, which were observed as a measure of normal background uptake, had a constant value with mean SUV uptake from baseline through week 20 (i.e., from the Infinia) was 2.1 ± 0.3 and for weeks 26 through 73 (i.e., from the Symbia) was 2.1 ± 0.1. Tumor response to therapy, over weeks 20 through 33, was not readily discerned from visual inspection or length measurements of the SPECT images alone when compared with SUV measurements. During this time interval, the patient underwent two courses of cyclophosphamide and topotecan at week 20 and two surgeries and four additional rounds of chemotherapy beginning at week 26. Due to the visual avidity and consistent length measurement at week 33, the patient’s disease was defined as refractory to treatment and referred for restaging. Additional chemotherapy and external beam radiotherapy were performed beginning at week 51. Starting from week 20, retrospective SUV analysis demonstrated a consistent decrease in SUV value in response to patient therapy; however, overall tumor length and [I-123] mIBG uptake were not readily apparent until week 40.

**Fig. 5:**
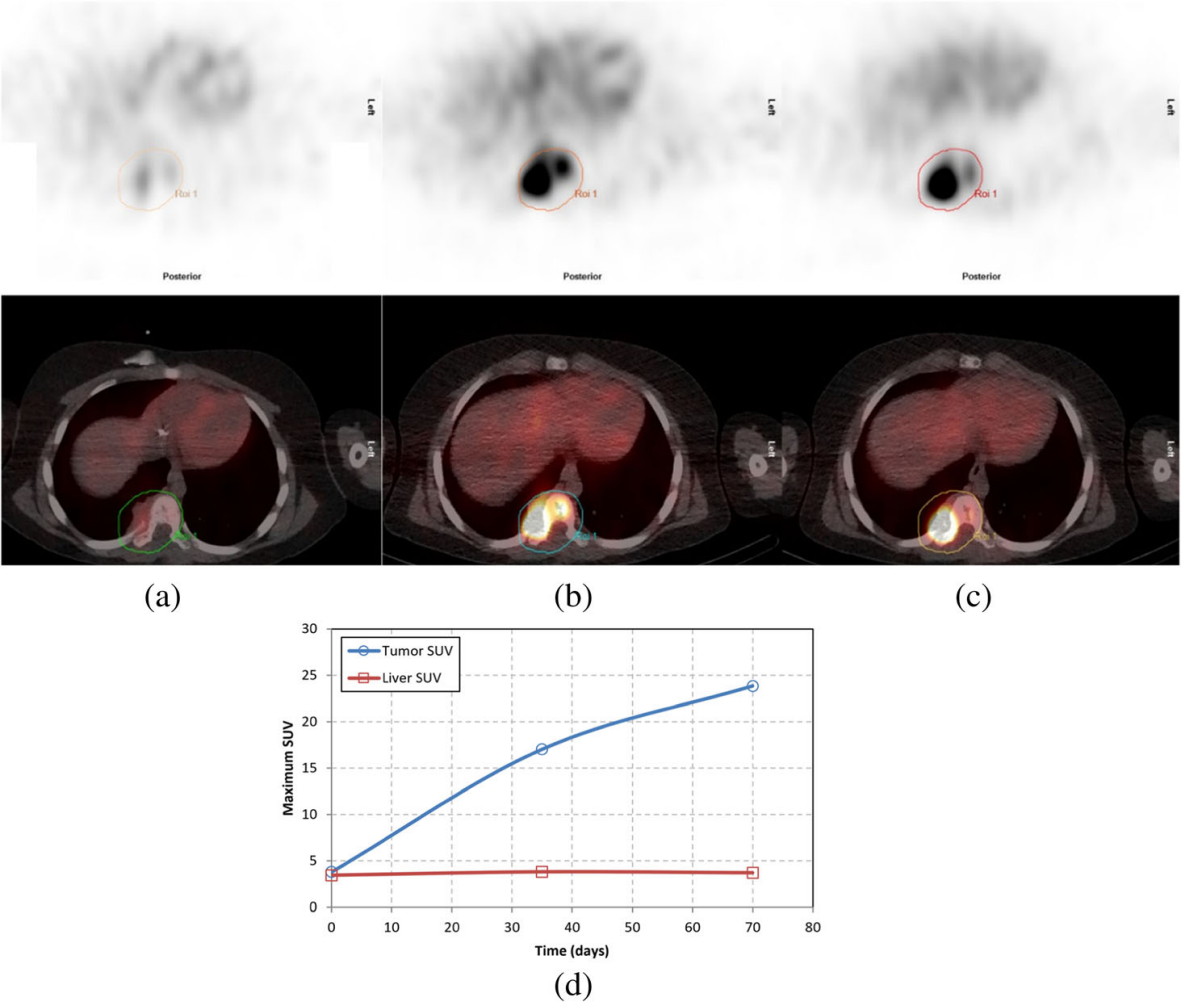
Example of quantitative SPECT analysis of an 11 year old boy with relapsed neuroblastoma undergoing treatment. **a** Baseline occurrence—SUVmax = 3.8; **b** 35 days since baseline and after 2 courses of chemotherapy—SUVmax = 17.1; finally (**c**) 70 days since baseline—SUVmax = 23.9. **d** Tumor SUV was plotted compared to background liver SUV. Patient subsequently underwent radiotherapy

Additionally, quantitative SPECT may be useful in the evaluation of recurrent or metastatic phaeochromocytoma, conditions with limited therapeutic options. High specific activity [I-131]-mIBG (Azedra) has received FDA approval for treatment of patients 12 and older with mIBG positive unresectable, locally advanced or metastatic phaeochromocytoma or paraganglioma [24, 25]. This treatment may result in reduction in antihypertensive medications and objective tumor response by radiographic criteria. Functional assessment of [I-123] mIBG uptake before and after therapy might add an objective functional measure of tumor response that could assist in evaluating the anti-tumor effect of this and other treatments (Fig. 5).

[I-123] mIBG is currently accepted as the best commercially available radiotracer for the staging of neuroblastoma, and it is included on most Children’s Oncology Group (COG) and international therapeutic protocols [26, 27]. Quantitative SPECT/CT [I-123] mIBG may provide objectivity in multi-institutional trials and central review; additionally, quantitative SPECT/CT may facilitate evaluation of neuroblastoma using radiomic techniques, as is being explored in quantitative DAT [28] and liver carcinoma [29] SPECT/CT.

This study was limited in scope to 1 academic clinical center. Additional work spanning multiple institutions and a variety of reader-observers should be useful for further definition of uptake of [I-123] mIBG in normal and neoplastic tissue. Assessment of clinical utility will require serial observations of many patients with neuroblastoma, preferably multi-institutional and in the context of group wide studies involving standardized protocols.

## Conclusion

The phantom results demonstrate accuracy for [I-123] SPECT on the order of 5–10%, consistent with other studies investigating [I-123] and other common radioisotopes. The patient examination results demonstrate consistent SUV calculation between two manufacturers’ SPECT scanners as generated by using a manufacturer-neutral quantitative SPECT software platform. Additionally, the results of this study demonstrate low intrapatient measurement variability for quantitative SPECT/CT analysis in a pediatric population with neuroblastoma. Quantitative SPECT/CT may offer the opportunity for objective analysis of tumor response using the conventional single photon–emitting agent [I-123] mIBG by normalizing the uptake to injected radiopharmaceutical dose and patient weight, as is done for PET.

## Data Availability

Data are stored in institutional password protected secured folders accessible through authors’ login credentials.
